# Beyond co-localization: inferring spatial interactions between sub-cellular structures from microscopy images

**DOI:** 10.1186/1471-2105-11-372

**Published:** 2010-07-07

**Authors:** Jo A Helmuth, Grégory Paul, Ivo F Sbalzarini

**Affiliations:** 1 Institute of Theoretical Computer Science, ETH Zurich, Universitätstrasse 6, 8092 Zürich, Switzerland; 2 Swiss Institute of Bioinformatics, ETH Zurich, Universitätstrasse 6, 8092 Zürich, Switzerland

## Abstract

**Background:**

Sub-cellular structures interact in numerous direct and indirect ways in order to fulfill cellular functions. While direct molecular interactions crucially *depend *on spatial proximity, other interactions typically *result in *spatial correlations between the interacting structures. Such correlations are the target of microscopy-based co-localization analysis, which can provide hints of potential interactions. Two complementary approaches to co-localization analysis can be distinguished: intensity correlation methods capitalize on pattern discovery, whereas object-based methods emphasize detection power.

**Results:**

We first reinvestigate the classical co-localization measure in the context of spatial point pattern analysis. This allows us to unravel the set of implicit assumptions inherent to this measure and to identify potential confounding factors commonly ignored. We generalize object-based co-localization analysis to a statistical framework involving spatial point processes. In this framework, *interactions are understood as position co-dependencies in the observed localization patterns*. The framework is based on a model of effective pairwise interaction potentials and the specification of a null hypothesis for the expected pattern in the absence of interaction. Inferred interaction potentials thus reflect all significant effects that are not explained by the null hypothesis. Our model enables the use of a wealth of well-known statistical methods for analyzing experimental data, as demonstrated on synthetic data and in a case study considering virus entry into live cells. We show that the classical co-localization measure typically under-exploits the information contained in our data.

**Conclusions:**

We establish a connection between co-localization and spatial interaction of sub-cellular structures by formulating the object-based interaction analysis problem in a spatial statistics framework based on nearest-neighbor distance distributions. We provide generic procedures for inferring interaction strengths and quantifying their relative statistical significance from sets of discrete objects as provided by image analysis methods. Within our framework, an interaction potential can either refer to a phenomenological or a mechanistic model of a physico-chemical interaction process. This increased flexibility in designing and testing different hypothetical interaction models can be used to quantify the parameters of a specific interaction model or may catalyze the discovery of functional relations.

## Background

A general biological principle states that cellular function results from the combined interactions of sub-cellular structures in space and time. Interactions typically manifest themselves through statistical dependencies in the spatial distributions of the involved structures. Here, we adopt this general definition and we understand *interaction *as the collection of all effects that cause significant (above the level predicted by a null hypothesis) correlations in the positions of the participating objects.

Over the last decades, advances in fluorescent markers have enabled probing interactions of sub-cellular structures in the microscope, either directly or indirectly. The *direct approach *relies on experiments that generate a signal upon the proximity required for molecular interaction. *Indirect approaches *are based on independently imaging two populations of interest, and searching for clues of interaction in their spatial distributions. This approach is based on the paradigm that spatial proximity (or *co-localization*) is a hallmark of many types of physical and chemical interactions between sub-cellular structures. If two or more structures interact, their spatial distributions hence appear correlated. The reverse, however, is not necessarily true. Presence or absence of significant co-localization does not imply presence or absence of interaction. The reason is that co-localization depends on the specific interaction mechanism: An unobserved third structure may act as a confounding factor (in the statistical sense), making the observed structures appear co-localized even though they do not interact. Furthermore, one can imagine interaction mechanisms that lead to spatial distributions with correlations that are not captured by simple co-localization measures. Hence, the interaction has to be statistically *inferred *from the data.

Such inference, however, entails a trade-off between the objectives of pattern discovery and statistical detection power. According to these objectives, two complementary approaches to co-localization analysis can be distinguished: Intensity correlation methods capitalize on pattern discovery [1], whereas object-based methods [2] emphasize detection power. Intensity correlation methods quantify correlations in the intensities of different color channels on individual pixels. Intensity correlation methods are straightforward to implement and use. The results, however, may be difficult to interpret since interactions need to be inferred from correlations in *intensity space*, which is sensitive to the blurring and noise inherent to microscopic imaging systems [3]. Object-based methods quantify the spatial relationships between sets of discrete objects. This requires reducing the image to a set of geometric objects using, e.g., image segmentation or fitting of structure models. Object-based approaches infer interactions from correlations in *physical space*, which allows constructing intuitive and simple co-localization measures, such as counting the number of overlapping objects [2].

The intensity-based approach is limited to interactions on a spatial scale on the order of the resolution of the microscope. While the object-based approach is not necessarily limited to any particular length scale (note that the localization accuracy for an isolated object is not limited by the spatial resolution of the microscope, but rather the signal-to-noise ratio [4-6]), a spatial scale is nevertheless assumed in practice. Many object-based co-localization methods rely on a hard threshold for the distances between objects in order to distinguish between "co-localized" and "not co-localized" for each individual pair of objects [2]. The choice of distance threshold greatly influences the types of interactions that can be reliably detected. The actual physical or chemical interaction between sub-cellular objects can be of short temporal duration and they can quickly separate thereafter. In such situations, high thresholds can increase the detection power, but only at the expense of increased false-positive rates. When interactions take place over long distances, the choice of threshold implicitly determines a range limit of the analysis.

Apart from fixing the interaction scale *a priori*, using a hard distance threshold also implies a binary distinction of pair-wise distances: either they are below the threshold and hence the objects are assumed to interact - or they don't. A co-localization percentage thus corresponds to an indirect measure for the preference of "interaction" over "non-interaction". This preference reflects the strength of the interaction. However, it also depends on the frequency of possible distances that the population of objects can assume.

More specifically, the cellular context in which the interactions take place is a confounding factor. A high co-localization percentage can, for example, be observed in a cell with densely packed sub-cellular structures of interest, irrespective of their interaction strength. This artifact needs to be considered in statistical tests [7] or corrected for in order to construct an interaction score [8].

Taken together, object-based approaches provide intuitive co-localization measures whose statistical interpretation, however, is not straightforward. Here, we establish a connection between co-localization and the notion of interaction as used in spatial statistics [9], namely the non-independence of the relative positions of objects under study. This is based on modeling the nearest-neighbor distance distribution between the observed objects. These distances are the result of interactions, measurement inaccuracies, and the geometry of the domain in which the objects are distributed. This modeling provides generic procedures for inferring interaction strengths and quantifying their statistical significance. Our approach helps formalizing design decisions in co-localization and interaction studies and shows how they translate to biological hypotheses. Standard object-based co-localization analysis is included as a special case, which makes explicit the connections between interaction and co-localization. After developing and characterizing the statistical interaction analysis framework, we exemplify its utility in a biological study of virus entry.

## Results and Discussion

### Basic scenario: co-localization analysis

We review the basic concepts of classical object-based co-localization analysis and its interpretation in terms of interactions.

**Figure 1:**
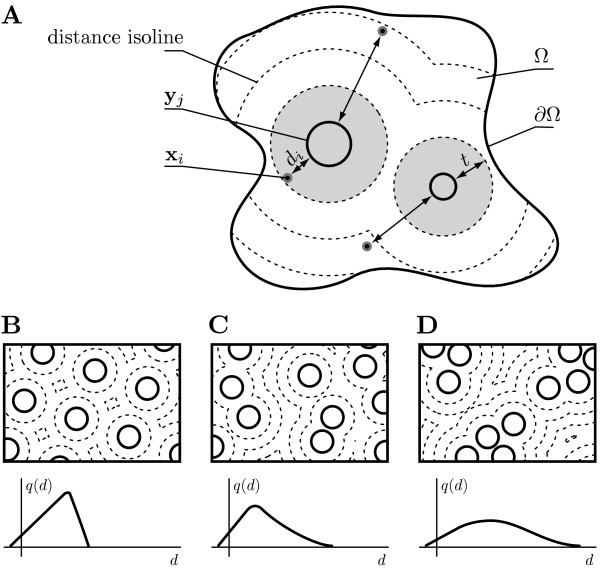
**Illustration of co-localization analysis and cellular context**. (A) Illustration of co-localization analysis based on nearest neighbor distances (arrows) between point-like objects  (dots) and circular objects  (solid circles). For all distances *d*, the state density *q*(*d*) is proportional to the total length of the *d*-isoline (dashed lines) in Ω. The expected co-localization in the absence of interactions, , is proportional to the area enclosed by the *t*-isoline (gray region). (B)-(D) Effect of the positioning of the objects *Y *on *q*(*d*), illustrating the influence of the cellular context.

Object-based co-localization measures are typically constructed for two sets of objects  and . These objects are located in a bounded region Ω ⊂ ℝ^*n *^with boundary ∂Ω and dimensionality *n *(usually 2 or 3; see Fig. 1). Each object *i *(*j*) is represented by a feature vector **x**_*i *_(**y**_*j*_) that comprises information about the object's position and, if available, its dimension and shape. These features vectors are extracted from image data by means of image segmentation or fitting of structure models.

Suppose one wishes to investigate the interaction between the objects in *X *and *Y*, one can define for each **x**_*i *_the distance to the nearest neighbor (NN) in *Y*,(1)

The function *d*(·) is a suitable distance function in feature space, for example the Euclidean distance between point-like objects or the minimum distance between outlines of more complex objects. A nearest-neighbor distance distribution *p*(*d*) can then be estimated from the set of distances . The classical *overlap *or *nearest-neighbor-distance co-localization measure C*^*t *^follows by counting [8]:(2)

where **1**(·) is the indicator function and *t *an application-specific distance threshold. The form of Eq. 2 implies assumptions about how the objects in *X *and *Y *interact. The interaction process is considered to be translation- and rotation-invariant since only the distance between interacting objects is taken into account. Based on this distance only two categories of positions of the objects in *X *are distinguished: either they are sufficiently close to any object in *Y *to be considered interacting, or they are not. Furthermore, objects in *X *interact with at most one object in *Y *and they do not experience the presence of any **y**_*j *_unless they cross the distance threshold *t*. The choice of *t *reflects an assumption about the length scale of the interaction to be detected.

Inferring interactions from an observed co-localization measure *C*^*t *^is not trivial since *C*^*t *^> 0 does not necessarily imply any interaction between the objects. This is because spatial correlations can also be caused by confounding factors, such as the cellular context {Ω, *Y*}. Even if the objects in *X *and *Y *do not interact there is a finite probability that any possible distance in an interval Δ*d *about *d*_*i *_is observed. We arbitrarily choose *Y *as a reference in order to compute the relative frequency of possible distances (*state density*) as:(3)

This density *q*(*d*) is determined by the positions, dimensions, and number density of the objects in *Y *(see Fig. 1). Independent random positions will result in a relatively wide density *q*(*d*) (Fig. 1C). With regularly placed objects *Y*, large distances do not occur (Fig. 1B). Clustering increases the frequency of long distances at the expense of short distances (Fig. 1D). Objects with large surfaces or a large number density give rise to shorter distances. In case there are interactions between the objects in *X *and *Y*, some of the possible distances are additionally favored over others, deforming the density *q*(*d*) to *p*(*d*).

The co-localization measure *C*^*t *^is, therefore, not sufficient to separate the contributions from the cellular context and the interactions. Information about the interactions is only contained in the *deviation *from an expected base-level in the absence of interactions. This base level, , is the co-localization measure that would be observed under the hypothesis *H*_0_: "no interaction" (obtained by letting *p*(*d*) = *q*(*d*) and numerical evaluation of the integral in Eq. 2). But how does a certain deviation from the base level  relate to interactions between the objects, and what deviations can be considered significant? We address this question in the following sections by generalizing co-localization analysis to interaction analysis. Ideally, an interaction score is independent of the cellular context and reflects variations of the interaction strength in a monotonous fashion. The first step toward constructing such a score is a precise definition of the term *interaction strength *in the context of an interaction model.

### Generalization: interaction analysis

*Spatial point process analysis *[9-11] is a standard statistical framework for studying the spatial distribution of interacting objects. Our interaction analysis is derived from the general binary Gibbs process with fixed number of objects. Its central component is an effective pair-wise interaction potential Φ(·). In many applications, "interaction" is an abstraction of the different effects that collectively cause an observed spatial pattern. Nevertheless, the mathematical form of the Gibbs process corresponds to physical models of interacting objects. The potential associates an energy level with each pair {*i*, *j*} of interacting objects. The probability density of the Gibbs process for two sets of interacting objects, *X *and *Y*, has the shape of a Boltzmann distribution:(4)

i.e., states with lower energy occur with higher probability. Eq. 4 implies mutual independence of the objects within the same set *X *or *Y*, in agreement with the assumptions formulated in the previous section. For nearest-neighbor interactions, the corresponding interaction potential is given by:(5)

where the function *ϕ*(*d*) specifies the distance dependence of the interaction.

Assume a cellular context {Ω, *Y*} is given. The probability density *p*(*X*|Ω, *Y*) for the potential in Eq. 5 then only depends on the *d*_*i*_. An inner sum over all *j*, as in Eq. 4, is then not required. The mutual independence within *X *allows factorizing *p*(*X*|Ω, *Y*) into terms that only depend on a single *d*_*i*_:(6)

where, unlike in Eq. 4, an explicit dependence of the potential on **x**_*i *_is no longer present.

The probability of observing a certain **x**_*i *_is proportional to exp (-*ϕ*(*d*_*i*_)). The probability of observing a certain *d*_*i*_, however, also depends on how frequently an arbitrary object **x **is a distance *d*_*i *_away from its NN in the given cellular context. This frequency is given by the state density *q*(*d*) as stated in Eq. 3. Straightforward calculations yield:(7)

The normalization constant *Z *(the *partition function*) renders *p *(*d*|*q*) a true probability density function.

So far, we have not specified any particular shape for the interaction potential *ϕ*(·), which can be a parametric or non-parametric model. A specific choice constitutes a hypothesis or assumption about the range, strength, and distance dependence of the interaction. These three aspects of the interaction are represented independently in our parameterization:(8)

ϵ is the strength, *f *encodes the shape, *σ *defines the length-scale, and *t *is a shift along the distance axis of the interaction potential. Using Eqs. 7 and 8 we find the joint probability density of observations *D*:(9)

This is the central class of models that we use to extend co-localization analysis to interaction analysis. All interaction models will be formulated as specific instances of such a model.

The assumptions underlying the simple overlap co-localization measure can, for example, be formalized in a specific interaction potential. Only two categories of distances (*d *<*t *and *d *≥ *t*) are distinguished (Eq. 2). This implies a step function for the shape *f*(*z*) of the interaction potential *ϕ*(*d*) (taking *σ *= 1):(10)

Using the integral definition in Eq. 2, the co-localization measure *C*^*t *^can then be expressed as a function of the interaction strength. Inserting Eq. 10 into Eq. 7 and Eq. 2 and solving for ϵ yields an estimator  of the model interaction strength:(11)

The quantity  corrects for the cellular context and, therefore, fulfills our requirements for a valid interaction score. Eq. 11 relates the purely descriptive co-localization measure *C*^*t *^to an interaction model between the objects in *X *and *Y*. It builds a bridge between patterns in the data (the cellular context summarized in *q *and the measure *C*^*t*^) and functional relationships (interactions) between sub-cellular components.

Whether an observed estimate  is indicative of the actual presence of an interaction, however, has to be addressed using statistical inference as presented in the following section.

### Hypothesis testing and power analysis for the step potential

In the parameterization of our interaction model (Eqs. 8 and 9), the presence of an interaction is equivalent to ϵ ≠ 0. Since  is an estimator, it is a random variable. Even if the hypothesis *H*_0_: "no interaction" is true, a non-zero  can occur with finite probability ( ≠ 0 does not imply ϵ ≠ 0). Inference about interactions requires finding a critical estimated interaction strength above which one can reject *H*_0 _on a prescribed significance level *α*.

This critical interaction strength is determined by the distribution of  under *H*_0 _(null distribution), which depends on the sample size *N*, *q*, and the prescribed *α*. Under *H*_0_, *C*^*t*^*N *is binomially distributed with parameters (, *N*). Hence, the critical *C*^*t *^can be computed from the (numerically) inverted cumulative distribution function of the binomial distribution. The corresponding critical  follows from Eq. 11.

**Figure 2:**
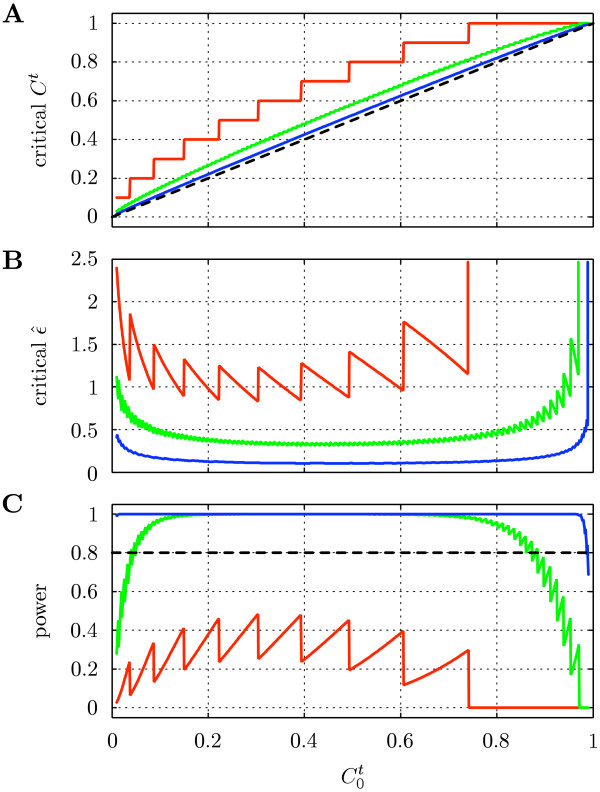
**Power analysis for a step potential**. Minimum *C*^*t *^(A) and  (B) that allows rejecting *H*_0_: "no interaction" (*α *= 0.05) as a function of the base-level . In A, the expected value of *C*^*t *^under *H*_0 _is indicated by a dashed line. (C) Statistical power (1 - *β*) for detecting interactions of a true strength ϵ = 1. Red, green, and blue lines correspond to *N *= 10, 100, and 1000, respectively, in all three panels.

The dependence of the critical *C*^*t *^and  on  and *N *is shown in Fig. 2A and 2B. It can be seen that the minimum significant excess over  varies only weakly with  (Fig. 2A). Obviously, large values of  in conjunction with small *N *do not allow rejecting *H*_0_, even if *C*^*t *^= 1. The critical value of  is highest at the two extremes of  and lowest for  ≈ 0.4 (Fig. 2B). As for *C*^*t*^, it can be seen that for large  and small *N *no finite  is sufficiently large to allow rejecting *H*_0_.

The curves in Fig. 2B show the decision of the statistical test based on the estimated interaction strength . A true interaction with a strength ϵ greater than this critical value does, however, not guarantee that it will always be detected by the test (type II error: *β*). Furthermore, a weak interaction may lead to unwanted rejection of *H*_0_. The behavior of the test critically depends on the effect size, which quantifies the departure from *H*_0_. Here, effect size refers to the true interaction strength ϵ = *a *> 0. The statistical "power" (1 - *β*) quantities the probability of rejecting *H*_0 _when *H*_1_: "*ϕ *= *ϕ*^st^, ϵ = *a*" is true. In Fig. 2C, the detection power for a true strength of *a *= 1 is shown as a function of . As expected from Fig. 2B, the power is low at the extremes of , eventually dropping significantly below the recommended value of 0.8, even for *N *= 100. Weak interactions are harder to detect, requiring larger sample sizes to yield a certain power.

In the design of experimental interaction studies, a key objective is to maximize the robustness and reliability of detecting effects of unknown size. Power can be increased by optimizing the experimental design or the subsequent statistical analysis. While increasing the sample size might be possible, controlling the cellular context is not feasible in most situations. Our analysis is based on the interaction model introduced in the previous section. It allows specifying different shapes *f*(·) and scales *σ *of the interaction potential. Power could potentially be increased by better modeling the interaction potential. In the next section, we thus quantify the influence of alternative model potentials on statistical power.

### Improving statistical power with non-step interaction potentials

Constructing statistical tests as described above requires assuming a specific shape and scale of the interaction potential. In the absence of prior knowledge, however, this model potential can be arbitrarily different from the true potential of the actual biological interactions under observation. Test statistics that are based on a model potential close to the real one may achieve greater power.

**Figure 3:**
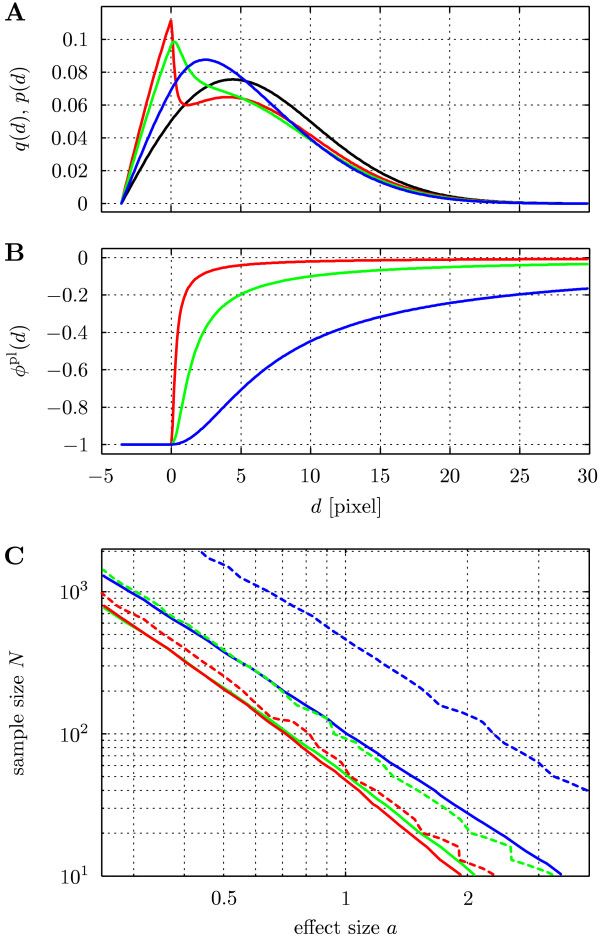
**Power analysis for non-step potentials**. (A) Black line: state density *q*(*d*) for *M *= 100 circular objects *Y *with radius *R *= 3.57 randomly placed in a square domain of size 200 × 200; *R *is chosen to yield a circle-covered area fraction of 0.1; Colored lines: resulting distance distribution *p*(*d*) for the three potentials shown in B. (B) Plummer potential (Eq. 12) with ϵ = 1 and varying scale parameter. (C) Monte-Carlo estimates of 80%-power isolines in the *N*-*a*-plane; dashed lines: tests based on *T*^st^, solid lines: tests based on *T*^pl^. Note that larger kinks in the dashed lines are due to the discreteness of *T*^st ^and are statistically significant. Colors in A-C indicate scale parameters of the true potential; red: *σ *= 0.2, green: *σ *= 1.0, and blue: *σ *= 5.0.

We quantify the influence of the discrepancy between the model and the true potential by considering a scenario where *N *objects {**x**_*i*_} are distributed in the square region Ω containing *M *randomly placed circular objects {**y**_*i*_} with identical radii *R*. Fig. 3A shows the corresponding state density *q*(*d*). The objects in *X *interact with the objects in *Y *according to the *Plummer potential *(with *t *= 0):(12)

This potential has an overall 1 = *d*-shape, but finite value and slope everywhere. The parameter ϵ again controls the interaction strength (potential depth). The parameter *σ *sets the length scale of the interaction (potential range) and allows gradually changing *ϕ*(*d*) from a step-like shape to a potential that causes significant attraction toward the objects in *Y *over large distances (see Fig. 3B).

For such more general potentials, algebraic expressions for  (such as Eq. 11 for the step potential) can in general not be derived. Statistical tests for the presence of interactions can nevertheless be constructed using a different statistic. Since Eq. 9 describes a member of the exponential family,(13)

is a sufficient test statistic for ϵ [12].

For a set of distances *D*, distributed according to Eq. 9 with *ϕ*(*d*) = *ϕ*^pl^(*d*), a test for the presence of interactions can thus be constructed based on  under *H*_0_: "no interaction", where the scale parameter *σ *is assumed to be known. The null-distribution can be approximated by i.i.d. Monte Carlo (MC) samples  (see Materials and Methods). An observed value of *T*^pl ^is then ranked among the . If it ranks higher than ⌈(1 - *α*)*K*⌉-th, *H*_0 _is rejected on the significance level *α *[12]. The statistical power of this test to reject *H*_0 _when *H*_1_: *ϕ *= *ϕ*^pl^, ϵ = *a*" is true, can be estimated with additional MC simulations: For a fixed effect size *a *> 0, one draws *N *distances *d*_*i *_from *p*(*d*), computes *T*^pl^, and conducts the test as described above [12]. This procedure is repeated many times and the fraction of tests rejected serves as an estimator of the power.

In order to quantify the influence of the model potential on statistical power, we test *H*_0 _against *H*_1 _and *H*_2_: "*ϕ *= *ϕ*^st^, ϵ = *a*" on data generated under *H*_1 _for varying *σ *(see Fig. 3B for the true interaction potentials under *H*_1_). Testing *H*_0 _against *H*_2 _makes use of the sufficient statistic , which is proportional to *C*^*t *^with *t *= 0. As opposed to *T*^pl^, this statistic only contains information about the signs of the *d*_*i *_and should thus yield a less powerful test.

Fig. 3C shows the number of samples required to reach 80% power as a function of the strength *a *of the true interaction potential. It can be seen that the power of a test based on the true interaction potential (solid lines) is higher than the power of a test based on a step potential (dashed lines). Moreover, this difference strongly increases with increasing potential range *σ*: for *σ *= 5 (blue lines) using the step model potential requires 4 times more samples. If the true potential is close to a step potential (*σ *= 0.2, red lines), both tests perform comparably well. Moreover, the figure also shows that interactions over longer distances are harder to detect. We therefore conclude that one needs to be careful when assuming a step potential (as implicitly done in traditional co-localization analysis). Controlling power requires prior knowledge about the interaction potential. Such prior knowledge can easily be included in the present framework by choosing *t*, *σ*, and *f*(*·*).

### Example: virus trafficking

The uptake and intracellular transport of virus particles is a complex process that involves temporary association with membrane receptors and multiple organelles of the endocytic machinery, such as early and late endosomes [13]. In many cases, fluorescence microscopy allows resolving the involved entities as discrete objects. This has previously motivated the use of object-based co-localization measures to quantify association kinetics and unravel infection pathways. Here, we show how the generalized framework of interaction analysis presented above can be applied in a practical experimental situation, and how it enables using a large toolbox of well-known statistical techniques.

We consider a set of 274 two-color fluorescence microscopy images of single HER-911 cells expressing the small GTPase Rab5 tagged with enhanced green fluorescent protein (EGFP), recorded in the green color channel. Rab5 is a regulator of clathrin-mediated endocytosis and a marker for early endosomes. These dynamic, lipid-bounded organelles are formed by invaginations of the plasma membrane. They are the first sorting compartment of clathrin-derived cargo [13]. Either fluorescently tagged Adenovirus serotype 2 (Ad2) or its temperature sensitive mutant (TS1) were recorded in the red color channel. Images were taken between 2 and 46 min post infection. The same data have already been used in a previous study [5]. Virus positions and endosome outlines were extracted from the images as described in the Materials and Methods section. Based on these object representations, the set *D *of virus-to-nearest-endosome distances and the state density *q*(*d*) were computed for each of the imaged cells.

Like Ad2, TS1 is known to enter the cell by clathrin-mediated endocytosis, but the mutation inhibits escape from endosomes [14,15]. This should be reflected in a deviation of the empirical distribution of observed distances *D *from the null distribution *p*(*d*) = *q*(*d*), which is stronger for TS1 than for Ad2. In our framework, this translates to a non-flat interaction potential between virus centroids and outlines of Rab5-positive endosomes.

Before modeling an interaction potential, we test *H*_0_: "*ϕ*(*d*) = 0" against *H*_1_: "*ϕ*(*d*) ≠ 0" for each imaged cell using a non-parametric statistical test (see Materials and Methods). This test does not assume any specific shape of the interaction potential, which allows detecting any type of interaction, albeit with reduced power. The results are summarized in Table 1. The fraction of cells for which *H*_0 _has to be rejected is significantly higher for TS1 than for Ad2, irrespective of the significance level and despite the on average smaller sample sizes *N*. However, Ad2 exhibits significant interaction with endosomes in half of the cells (*α *= 0.05).

**Table 1:** Results of non-parametric statistical tests for interaction in the virus trafficking data.

	#cells	*p *< 0.05	*p *< 0.01	*N*
Ad2	135	70 (52%)	25 (19%)	180 ± 50
TS1	139	128 (92%)	100 (72%)	157 ± 59

First column: number of cells analyzed; second and third columns: number and percentage of cells for which *H*_0 _was rejected on the indicated significance levels; forth column: mean and standard deviation of the observed number of virus particles per cell.

**Figure 4:**
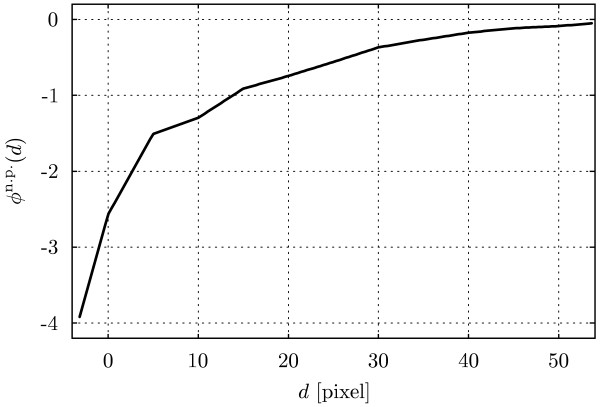
**Non-parametric estimate of the interaction potential**. The non-parametric estimate of the interaction potential based on all imaged cells.

These results indicate that the interaction potential is non-zero for many cells. They do not, however, permit any conclusions about the shape or strength of the interaction potential, for which, in addition, no prior information is available. We therefore apply a non-parametric estimation procedure for the interaction potential to get a sketch of its strength and distance-dependence. Subsequently we can specify and identify parametric potentials. Ignoring, for now, possible variability between cells and virus types, we pool all data and estimate a common non-parametric potential  (see Materials and Methods). The estimated  is shown in Fig. 4. Its shape is notably different from a step function. The slow decay suggests that viruses interact with endosomes over distances of about 10 pixels (1 *μ*m) from their center.

The estimated non-parametric potential serves as a template for the shape of parametric models. Parametric potentials can be identified more robustly from sets of observed distances of individual cells. This allows correlating their parameters with co-variates such as the virus type or the time at which a cell was imaged after infection. We consider four different potentials, two that resemble the shape in Fig. 4 (*Hermquist *and *Linear type 1*) and two that are generalizations of the step potential with a plateau below *d *= 0 (*Linear type 2 *and *Plummer*). For all potentials, we fix the threshold to *t *= 0. Definitions of the potential shapes *f*(·) are given in the Materials and Methods section.

The parameters of the potentials are found by maximum likelihood estimation (MLE). In order to exclude cell-to-cell variations of the potential range, we do not determine the pairs (ϵ_*k*_, *σ*_*k*_) for each cell separately. Rather, we estimate for a given potential a single scale parameter *σ*_*k *_= σ* common to all cells, while the interaction strengths ϵ_*k *_may vary between cells. The resulting (*N*^cells ^+ 1)-dimensional estimation problem is solved with a nested ML algorithm (see Materials and Methods). The common scale  and the maximum of the pooled log-likelihood l* for the four potentials are reported in Table 2. As a reference, the values are also given for a step potential with distance threshold *t *= 0.

**Table 2:** Comparison of estimated scale parameters of interaction potentials for the virus trafficking data.

		max l*	rank
Hermquist	3.96	-1.2247·10^5^	1
Linear, type 1	4.14	-1.2362·10^5^	2
Linear, type 2	6.61	-1.2427·10^5^	4
Plummer	1.15	-1.2374·10^5^	3

Step	(*t *= 0)	-1.2632·10^5^	5

Scale parameters  of potentials as found by maximum-likelihood estimation, and the corresponding maximized pooled log-likelihoods max l* for the different potentials (Eq. 17.)

**Figure 5:**
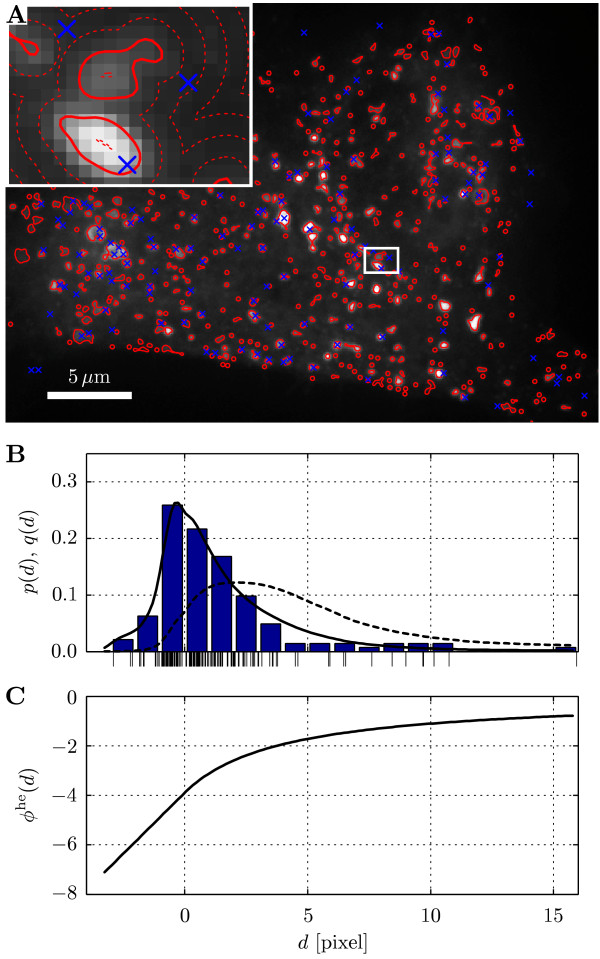
**Interaction analysis applied to virus trafficking**. Interaction analysis for a single cell infected with TS1, imaged 27 min post infection. (A) Imaged endosomes (Rab5-EGFP) with overlaid outlines (solid red lines) and virus centroid positions (blue crosses, virus channel not shown). Nearest-endosome-distance isolines (dashed red lines) are shown in the magnified inset. (B) State density *q*(*d*) for the shown cell (dashed black line), observed virus-to-nearest-endosome distances (marks and histogram, *N *= 143), and estimated distance distribution from the model *p*(*d*) (solid black line). (C) Estimated Hermquist potential ( = 3.90,  = 3.96) of the interactions between viruses and nearest endosomes.

The potentials are ranked according to their log-likelihood. It can be seen that the step potential is outperformed by all others. This remains unchanged even if one compares Akaike or Bayesian information criteria, which take into account the smaller number of free parameters. With a difference in log-likelihood of > 10^3 ^to second-best fit, the Hermquist potential is by far the best fit. It is also subjectively most similar to the non-parametric potential identified above. Fig. 5 shows an example of an imaged cell, infected with TS1, together with the empirical and estimated distance distributions and the corresponding Hermquist potential. The images of Ad2-infected cells are visually indistinguishable from those of TS1-infected cells and are hence not shown. Despite fitting only one independent parameter (σ* is fixed from the estimate over all cells), the estimated model distribution captures the features of the data remarkably well.

**Figure 6:**
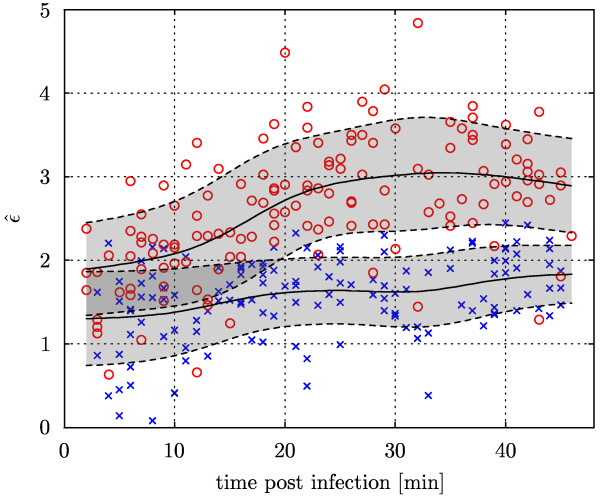
**Time-resolved interaction analysis of the trafficking of two strains of viruses**. Estimated strength of a Hermquist potential (scale σ* = 3.96) for the interaction between endosomes and virus particles versus the time post infection. Red circles: TS1; blue crosses: Ad2. The time course of the mean (solid lines) and the ± 1 standard deviation interval (shaded bands) are estimated using a Nadaraya-Watson kernel estimator with bandwidth of 5 min.

The estimated interaction strength  of the Hermquist potential varies within and between the two groups of infected cells. The within-group variability comprises statistical fluctuations and natural variations between cells. Since virus internalization and transport is a dynamic process, the time at which a cell was imaged (time post infection) is a further source of in-group variability. Fig. 6 shows the estimated interaction strength of a Hermquist potential for all cells infected with Ad2 (blue crosses) and TS1 (red circles) as a function of the time post infection. Throughout the observation period, the interaction strength for TS1 is significantly larger than that for Ad2, confirming the trend reported in Table 1. Furthermore, a temporal maximum of the interaction strength is apparent for TS1, while for Ad2 no significant variation over time can be resolved. These results indicate that TS1 and Ad2 use different uptake pathways or exhibit significantly different escape kinetics from Rab5-positive endosomes.

## Conclusions

We have introduced a statistical inference framework for robustly estimating interaction parameters from experimentally observed object distributions.

This allowed establishing a connection between spatial co-distributions of objects and interaction, by formulating the object-based interaction analysis problem in a spatial statistics framework based on nearest-neighbor distance distributions. The present framework provides generic procedures for inferring interaction strengths and quantifying their statistical significance. Standard object-based co-localization analysis is included as a limit case, making explicit the connections between the present framework and more classical approaches.

In the present framework, two novel key quantities emerge: (i) the state density *q*(*d*), which is the distribution of nearest-neighbor distances expected under the null hypothesis of no interaction, and (ii) the interaction potential *ϕ*(*d*), which defines the strength and distance dependence of the interaction. We have shown that classical co-localization analysis amounts to estimating the parameters of a step potential. This requires a notion of "inside" and "outside", either naturally defined by the physical extent of the objects or imposed through the step function's distance threshold. For point-like objects, or weak correlations between object positions, the choice of distance threshold is arbitrary.

This limitation can be relaxed by affording more general shapes of the interaction potential, which naturally extends co-localization analysis to (spatial) co-distribution analysis without requiring any additional assumptions. The additional flexibility allows capturing information about a wider range of sub-cellular interactions. This was demonstrated by statistical power analysis of the classical and generalized measures. Our results highlight that the probability of detecting an interaction strongly depends on the cellular context. We furthermore illustrated the influence of the range of an interaction on its detectability. Test statistics that include knowledge about the shape of the true interaction potential can greatly reduce the number of samples required to achieve a certain target power. Physico-chemical models might provide such prior knowledge. Alternatively, a non-parametric phenomenological potential can be estimated from the data as demonstrated here. This potential can then serve as a template for the parametric potentials used in subsequent analyses. In addition, the present framework enables comparison of the likelihoods of different hypothetical physico-chemical interaction models directly on the original image data.

The present approach enables applying a wide range of established statistical tools for analyzing experimental data, from parameter identification to model selection. This workflow was illustrated by studying the spatial patterns of endosomes and viruses infecting live human cells. In this case study, the experimental data were very well explained using only a single free parameter per cell. Among the five potentials considered, the step potential (corresponding to the classical co-localization measure) was worst in explaining the data. This highlights the benefit of the present method over classical co-localization analysis. Moreover, the fitted potentials provided additional quantitative readouts that could be used in subsequent machine learning analyses.

For simplicity the case study was done on 2D projections of 3D images. The presented approach, however, is equally applicable in three dimensions without any changes, provided three-dimensional object detection and segmentation is available. Projecting the data into two dimensions alters the estimated potentials (as it also does for any other co-localization measure), since it distorts both the distance data *D *and the state density *q*(*d*). We empirically found that the strengths of the potentials estimated from the projected 2D data may be *smaller *than those estimated directly on the raw 3D data (data not shown). Although all distances *D *are systematically reduced by the projection, this effect is overcompensated by the non-linear distortion of *q*(*d*), which is strongest for intermediate distances, but negligible for very small and large distances. Besides projection artifacts, errors in the image processing may also influence the estimated co-localization measures. Depending on the accuracy of the image segmentation method used, object sizes can be under- or overestimated, or entire objects can be missed altogether. This problem is inherent to all forms of co-localization or distribution analysis. We have assessed the sensitivity of our method with respect to image segmentation errors by successively eroding or dilating the endosomes from the presented case study. The results show that the mean of the estimated strength of the Hermquist potential remains unaffected, yet the variance of the estimate increases for strong erosion when entire endosomes start to be missed (data not shown). This robustness of the present method is due to the state density *q*(*d*) correcting for size errors. The classical co-localization measure, naively corrected for the cellular context by subtracting the amount of unspecific co-localization *C*_0_, significantly changes when under- or over-estimating object sizes. For strong erosion, leading to very small and frequently missing objects, it even drops to a meaningless value of zero (data not shown). Since image segmentation errors are always present in practical applications, we consider the robustness of our method one of its major advantages over classical measures.

The presented framework is limited by the same assumptions that also underlie classical co-localization analysis: (i) spatial homogeneity and (ii) isotropy of the interaction within the observation window, and (iii) exclusively nearest-neighbor interactions between objects of different classes. Assumption (i) is, e.g., violated if large areas of the analyzed images do not contain any objects. In this case, estimation of *q*(*d*) is not robust. Assumption (iii) imposes limits on admissible distances between objects: If objects *X *are attracted toward objects *Y*, the distances between the objects within the set *Y *need to be larger than the typical interaction range.

All of these limitations could be relaxed by using position-dependent interaction potentials or allowing for many-body interactions as described by general Gibbs processes. Considering such processes, however, is theoretically and numerically challenging. The presented framework could also be extended by including additional confounding factors, such as imaging artifacts causing spurious co-localization. Temporal plasticity of interactions, cell-to-cell variations, and experiment-to-experiment variations could be accounted for through additional co-variates (time, cell index, experiment index) in the statistical model. Already in its present form, the statistical framework can be used to test more general hypotheses, such as "interactions are stronger in strain A than in strain B".

The interpretation of fitted potentials is limited to their relative strengths. In the absence of a mechanistic or physical model of the process that has created the observed spatial pattern, biophysical interpretation of the identified parameter values is difficult or misleading. This is because the fitted interaction potentials reflect the collection of all intracellular phenomena that lead to the observed point pattern. Interestingly, however, a relation between the steady-state distribution of a diffusion process with added deterministic forces and the distribution of the Gibbs process (Eq. 4) exists: If the deterministic force acting between the diffusing objects is given by -∂*ϕ*/∂*d*, the two distributions become identical (in appropriate units). This fact points a possibility of connecting fitted interaction potentials with biophysical processes.

## Methods

### Image acquisition and processing

Endosomes and virus particles were imaged with a high-resolution spinning disk confocal microscope (NA 1.35, 100× objective plus additional 1.6× lens, 100 nm pixel size) as described [5]. We acquired *z*-stacks of 8 images each with a 400 nm *z*-spacing. Stacks were maximum projected prior to image analysis. Endosome outlines were represented as piece-wise linear closed splines in the focal plane. Outlines were estimated from images using a specialized model-based image analysis technique [5], yielding sub-pixel localization accuracy and precision. Virus particles were modeled as points and represented by estimated intensity centroid positions [6]. Prior to distance measurement, relative shifts between virus and endosome positions due to chromatic aberration were corrected using an empirical calibration function [5,16]. The boundary ∂Ω of the region Ω was defined as the cell boundary. An approximation of it was found by low-pass filtering and thresholding of the endosome images.

### Measuring q(d)

The state density *q*(*d*) was determined from the objects {**y**_*i*_} contained in the region Ω. Positions **x **in Ω were sampled exhaustively on a uniform Cartesian grid with spacing *h *= 0.25 pixel. For each **x**, the distance *d*_*i *_to the nearest neighbor in *Y *was computed. Using this finite sample of distances *D *= {*d*_*i*_}_*i*_, an approximation of *q*(*d*) was found by Gaussian kernel smoothing density estimation using the MATLAB (The MathWorks, Inc.) function ksdensity.m with default settings.

### Test for interaction

Following [12], a non-parametric test for interaction was constructed using the distance counts(14)

in *L *= 20 equi-sized bins defined by *L *+ 1 strictly increasing thresholds *t*_*l *_that span the entire non-zero range of *q*(*d*) for a given cell. First, a Monte Carlo sample  from the null distribution of **T **was obtained by sampling *N *= |*D*| distances *d*_*i *_from *q*(*d*), computing **T**_*k*_, and repeating this procedure *K *times. This sample allowed approximating the expectation **E**_0_(**T**) and co-variance matrix **Cov**_0_(**T**) of the null distribution. The test statistic *U *was defined as(15)

Second, **T **and *U *were computed for the set *D *of observed distances. *U *was then ranked among the  obtained from an additional Monte Carlo sample , generated as described above. If it ranked higher than ⌈(1 - *α*)*K*⌉-th, *H*_0 _was rejected on the significance level *α*.

The parametric tests used in sections "Hypothesis testing and power analysis for the step potential" and "Improving statistical power with non-step interaction potentials" followed a simpler protocol. The ranking was directly performed among the scalar test statistics *T*^st ^and *T*^pl^, avoiding the detour via *U*. A priori estimation of the expectation and variance of *T*^st ^and *T*^pl ^was therefore not required.

### ML estimation of potentials

For a given potential *ϕ*, the log-likelihood of its parameters Θ given the observations *D*_*k *_in cell *k *is:(16)

Simultaneous estimation of the common scale σ* and independent strengths ϵ_*k *_of a set of *N*^cells ^cells was done by maximizing the pooled log-likelihood:(17)

with respect to the parameters {Θ_*k*_} = {(ϵ_*k*_, σ*)}. This was done by numerically maximizing (using Nelder-Mead simplex) the sum of maxima *l*((ϵ_*k*_, σ*)|*D*_*k*_, *k*) with respect to σ*.

The piece-wise linear non-parametric potential  was defined as a weighted sum of kernel functions *κ*(·) centered on the support points *d*_*p*_:(18)

*P *= 21 support points *d*_*p *_were distributed between -5 and 95 with constant spacing *h *= 5 pixel. Setting *w*_*P *_= 0 enforced  = 0 for all *d *≥ 95. Setting *ϕ *= *ϕ*^n.p. ^the remaining weights were estimated by numerically maximizing (using CMA-ES) the penalized joint log-likelihood [17]:(19)

with respect to Θ = (*w*_1_,...,*w*_*P*-1_). Smoothness of *ϕ*^n.p. ^was controlled by the parameter *s *= 2. The quadratic penalty in Eq. 19 corresponded to a Gaussian prior with zero mean and standard deviation *s *on the differences *w*_*p *_- *w*_*p*+1_.

### List of parametric potentials

Potentials were parameterized as *ϕ*(*d*) = ϵ*f*((*d *- *t*)/*σ*) with interaction strength ϵ, length scale *σ*, and threshold *t *= 0. Their shapes *f*(·) were defined as:

• Hermquist potential:(20)

• Linear potential, type 1:(21)

• Linear potential, type 2:(22)

• Plummer potential: defined in Eq. 12.

### Implementation

All software was implemented in MATLAB version 7.9 (The Mathworks, Inc.) and run on a 2.66 GHz Intel Core2 Duo machine. Estimation of two-parameter potentials (Eqs. 12 and 20 to 22) took a few milliseconds per cell. Computation of *q*(*d*) took about one second. This time, however, strongly depended on the sampling resolution used. The non-parametric test for interaction took about half a second per cell. The time needed to estimate the common scale parameter for all cells was around ten minutes. A constantly updated version of the developed software is freely available from the web site of the authors http://www.mosaic.ethz.ch/Downloads. The MATLAB functions, scripts, and sample data at the time of writing are contained in additional file 1.

## Authors' contributions

JAH and GP developed the theory and analyzed the virus trafficking data. JAH designed, conducted, and analyzed numerical experiments and drafted the manuscript. GP participated in designing and analyzing the numerical experiments and helped in writing the manuscript. IFS participated in designing the theory and numerical experiments, helped writing and editing the manuscript, and coordinated the project. All authors read and approved the final manuscript.

## Supplementary Material

Additional file 1 **MATLAB source code**. ZIP archive containing the MATLAB source code for potentials, likelihood functions, and statistical tests, as well as sample scripts and sample data at the time of writing. Click here for file
